# Second primary malignancies in postpolycythemia vera and postessential thrombocythemia myelofibrosis: A study on 2233 patients

**DOI:** 10.1002/cam4.2107

**Published:** 2019-06-07

**Authors:** Barbara Mora, Elisa Rumi, Paola Guglielmelli, Daniela Barraco, Margherita Maffioli, Alessandro Rambaldi, Marianna Caramella, Rami Komrokji, Jason Gotlib, Jean Jacques Kiladjian, Francisco Cervantes, Timothy Devos, Francesca Palandri, Valerio De Stefano, Marco Ruggeri, Richard T. Silver, Giulia Benevolo, Francesco Albano, Chiara Cavalloni, Daniela Pietra, Tiziano Barbui, Giada Rotunno, Mario Cazzola, Alessandro Maria Vannucchi, Toni Giorgino, Francesco Passamonti

**Affiliations:** ^1^ Hematology, Department of Medicine and Surgery University of Insubria Varese Italy; ^2^ Department of Hematology Oncology Fondazione IRCCS Policlinico San Matteo, Università di Pavia Pavia Italy; ^3^ CRIMM‐Centro Ricerca e Innovazione delle Malattie Mieloproliferative, Department of Experimental and Clinical Medicine Azienda ospedaliera‐Universitaria Careggi, University of Florence Florence Italy; ^4^ Hematology and BMT Unit ASST Papa Giovanni XXIII, University of Milan Bergamo Italy; ^5^ Ospedale Niguarda Cà Granda Milano Italy; ^6^ Moffit Cancer Center Tampa Florida; ^7^ Stanford University Palo Alto California; ^8^ Hôpital Saint‐Louis et Université Paris Diderot Paris France; ^9^ Hospital Clínic, IDIBAPS, University of Barcelona Barcelona Spain; ^10^ Department of Hematology, University Hospitals Leuven and Laboratory of Experimental Transplantation, Department of Microbiology and Immunology KU Leuven Leuven Belgium; ^11^ Policlinico S. Orsola‐Malpighi Bologna Italy; ^12^ Università Cattolica del Sacro Cuore Roma Italy; ^13^ Ospedale S. Bortolo Vicenza Italy; ^14^ Weill Cornell Medical College New York; ^15^ Centro Oncologico Ematologico Subalpino (COES) Torino Italy; ^16^ Università di Bari Bari Italy; ^17^ FROM Research Foundation ASST Papa Giovanni XXIII Bergamo Italy; ^18^ Biophysics Institute, National Research Council of Italy Milan Italy; ^19^ Department of Biosciences University of Milan Milan Italy

**Keywords:** JAK inhibitors, second malignancy, secondary myelofibrosis

## Abstract

Patients with myeloproliferative neoplasms (MPN) are known to have higher incidence of nonhematological second primary malignancies (SPM) compared to general population. In the MYSEC study on 781 secondary myelofibrosis (SMF) patients, the incidence of SPM after SMF diagnosis resulted 0.98/100 patient‐years. When including non‐melanoma skin cancers (NMSC), the incidence arose to 1.56/100 patient‐years. In SMF, JAK inhibitor treatment was associated only with NMSC occurrence. Then, we merged the MYSEC cohort with a large dataset of PV and ET not evolving into SMF. In this subanalysis, we did not find any correlation between SPM and SMF occurrence. These findings highlight the need of studies aimed at identifying MPN patients at higher risk of SPM.

Polycythemia vera (PV) and essential thrombocythemia (ET) are myeloproliferative neoplasms (MPN) that can progress to post‐PV (PPV) myelofibrosis (MF) and post‐ET (PET) MF (from now on referred to as secondary myelofibrosis—SMF) with a progressive clinical phenotype.^1^ Among 20,250 MPN patients included in the Surveillance, Epidemiology, and End Results Program (SEER) database,^2^ the 10‐year cumulative incidence of nonhematological second primary malignancies (SPM) was 12.7%, significantly higher than that expected in the general US population. Information on development of SPM in SMF is scant.

Objectives of this study are to establish SPM incidence in SMF, to investigate potential relationship between SPM and SMF occurrence in PV and ET, and to address potential effect of JAK inhibitors (JAKi) on SPM occurrence in SMF. For these purposes, we evaluated the MYSEC cohort 3 of 781 SMF and the Pavia cohort of 611 PV and 841 ET patients not evolved into SMF during a median follow up of 4.6 years (range, 0.1‐39.7). PV, ET, and SMF diagnoses were reviewed according to the WHO and the IWG‐MRT criteria, respectively. The study was approved by the Review Board of each Institution and conducted in accordance with the Declaration of Helsinki. We performed time‐to‐event analysis with Cox regression models. Pre‐ and post‐SMF periods were treated considering SMF as a time‐dependent state. Likewise, JAKi treatment was considered a time‐dependent covariate present from the date of drug start. We defined SPM all malignancies except myelodysplastic syndromes, acute leukemias, carcinomas in situ, breast fibroadenomas, superficial bladder carcinomas, and nonmelanoma skin cancers (NMSC). SPM* included SPM and NMSC.

In the MYSEC cohort, within a median follow‐up of 14.8 years (range, 0.9‐46) from PV/ET diagnosis, 55 patients (7%) developed SPM. Among these, eight did not have the SPM date available and were excluded from the time‐dependent analysis. Twenty‐two (46.8%) developed a SPM during the ET/PV phase and 25 (53.2%) after SMF transformation. SPM subtypes are described in Figure 1.

**Figure 1:**
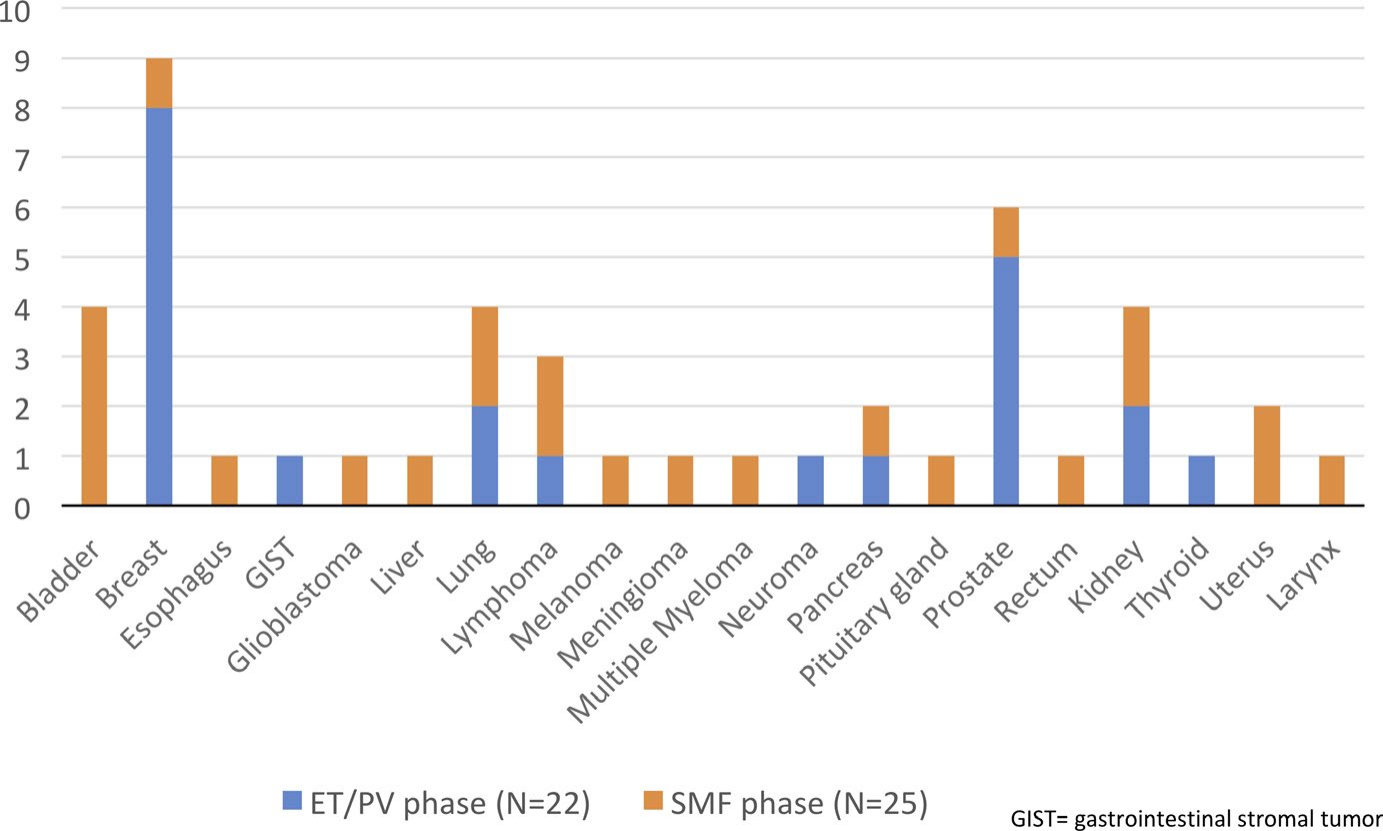
Distribution of secondary primary malignancies (SPM) in the MYSEC cohort

The incidence of SPM after SMF diagnosis was 0.98/100 patient‐years. There was a trend of association between male gender and SPM occurrence (*P* = 0.055). No statistically significant differences in clinical presentation, driver mutations, karyotype, bone marrow fibrosis, and MYSEC‐PM strata at the time of SMF diagnosis were found within SMF patients with and without SPM.

When including NMSC (SPM* group), we found 77 (9.9%) cases, 67 of them with date of diagnosis available: 26 (38.8%) during the ET/PV phase and 41 (61.2%) after SMF transformation. The incidence of SPM* after SMF diagnosis was 1.56/100 patient‐years. No significant differences in terms of clinical phenotype and genotype were found within SMF patients with and without SPM*.

Merging the MYSEC and the Pavia cohorts allowed us to evaluate the impact of SMF transformation on the SPM occurrence (treated as time‐dependent variable) in PV and ET. The incidence of SPM resulted not significantly different between patients who evolved into SMF (MYSEC cohort) and those who did not (Pavia cohort) (*P* = 0.06, Figure 2A). Conversely, the incidence of SPM* was significantly higher in patients who evolved into SMF (*P* = 0.002, Figure 2B), also when adjusted for age at the time of PV/ET (HR: 1.56, 95%CI: 1.0‐2.4; *P* = 0.04).

**Figure 2:**
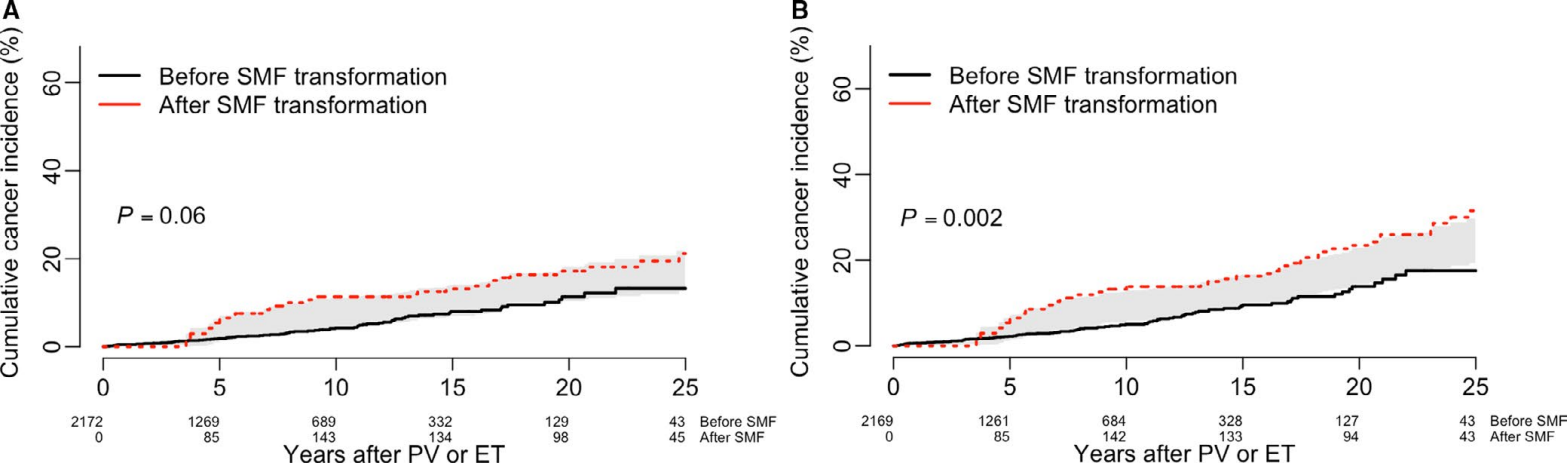
Cumulative incidence of second primary malignancies in patients with essential thrombocythemia (ET) and polycythemia vera (PV) with or without transformation into secondary myelofibrosis (SMF). Data are from 2233 patients with PV and ET, excluding (A) or including (B) nonmelanoma skin cancers

Finally, we assessed the effect of JAKi treatment on the occurrence of SPM in 151 patients of the MYSEC database: 111 received ruxolitinib, 10 fedratinib, 11 momelotinib, one XL019, and 18 a JAKi sequence. Overall, four patients (2.6%) developed SPM (all treated during SMF phase) within a median time of JAKi exposure of 1.2 years (range, 0.2‐2.2): one case each of renal, liver, rectal, and pancreatic cancer. We did not find any correlation between JAKi (treated as time‐dependent variable) and occurrence of SPM (log‐rank *P* = 0.34). Of interest, none of the two SMF who had lymphomas had been treated with JAKi. On the other hand, on extending the analysis to SPM*, eight cases (5.3%) were diagnosed. We found a significant correlation between JAKi and occurrence of SPM* in SMF (*P* = 0.02). This was confirmed even adjusting for the SMF subtype and for age at SMF diagnosis (HR: 2.4; 95% CI: 1.1‐5.4; *P* = 0.03).

A clear correlation between cytotoxic treatments and SPM occurrence has never been clearly demonstrated in MPN. Hydroxyurea treatment is associated with skin damage and with NMSC. However, a recent study reported a significantly higher number of SPM in MPN patients who had received no prior therapy, as compared with patients who received monotherapy or multiple therapies.^4^ The wide use of JAKi and their effect on immunity control has raised the issue of SPM in patients under treatment. A higher incidence of NMSC has been documented in PV receiving ruxolitinib (especially in those who received hydroxyurea first).^5^ Our data confirm this relationship highlighting the need for treating physicians to monitor cutaneous cancers before and during JAKi. We did not find any lymphoma in our cohort of 151 JAKi‐treated patients, differently from a recent report on 69 patients, however, with a longer 25‐month JAKi exposure.^6^

In conclusion, this study provides evidence that in PPV and PET MF the incidence of SPM and SPM* is about 1.0 and 1.5/100 patient‐years, respectively. There was no evidence of association between JAKi treatment and SPM development, with the exception of NMSC occurrence. Finally, we showed that in patients with PV or ET the occurrence of SMF is not associated with that of SPM, leaving the two events pathogenetically independent. The higher occurrence of NMSC we found is probably related to the use of hydroxyurea first or JAKi in the last times. These findings highlight the need of studies aimed at identifying patients at higher risk of second primary malignancies.
